# Transoral surgery with Thunderbeat© for hemangioma of the tongue base: A novel procedure. Case report

**DOI:** 10.1016/j.ijscr.2024.109385

**Published:** 2024-02-10

**Authors:** Carmelo Saraniti, Davide Burrascano, Iara Comincini, Anna Martorana, Barbara Verro

**Affiliations:** a Unit of Otorhinolaryngology, Department of Biomedicine, Neuroscience and Advanced Diagnostic, University of Palermo, 90127 Palermo, Italy; b Unit of Otorhinolaryngology - Head and Neck Surgery, ASST Spedali Civili of Brescia, Department of Medical and Surgical Specialties, Radiological Sciences, and Public Health, University of Brescia, Brescia, Italy; c Pathology Unit, Department of Health Promotion Sciences Maternal and Infantile Care, Internal Medicine and Medical Specialties, University of Palermo, 90127 Palermo, Italy

**Keywords:** Hemangioma, Tongue base lesion, Cavernous hemangioma, Transoral ultrasonic surgery, Ultrasonic surgical procedures

## Abstract

**Introduction:**

Hemangiomas are the most common benign tumors of the head-neck region in children and mainly affect the face, oral mucosa, lips, and tongue. The base of tongue is an extremely rare site of involvement. The incidence is higher in women and occur more frequently in infants and childhood.

**Presentation of case:**

We present a rare case of cavernous hemangioma of the base of tongue in a 70-year-old male patient surgically removed by Transoral Ultrasonic Surgery (TOUSS). 1-year follow up didn't show sign of recurrence.

**Discussion:**

Hemangiomas are benign proliferations of endothelial cells common in the head and neck. The etiology is uncertain: an imbalance in angiogenesis related to substances such as vascular endothelial growth factor (VEGF) and basic fibroblast growth factor (BFGF) with uncontrolled proliferation of vascular elements is proposed. It can be asymptomatic or, when affecting the tongue, lead to difficulty swallowing, pain, bleeding and dyspnea.

**Conclusion:**

This case report aims to stress that hemangioma should be considered in differential diagnosis in case of richly vascularized tongue base lesion, also in adult population. It would like to highlight the role of transoral ultrasonic surgery (TOUSS), which is able to achieve the same advantages as TORS with lower costs and shorter learning curve.

## Highlights

• Hemangiomas are the most common benign tumors of the head-neck region in children;
• Cavernous hemangioma of the base of tongue is very rare, especially in adult men;
• Transoral ultrasonic surgery is minimally invasive transoral surgical technique;
• Thunderbeat© combines ultrasound and radiofrequency;
• Thunderbeat© ensures vessel sealing, fast cutting, minimal adjacent thermal damage.

## 1 Introduction

The hemangioma is a benign proliferation of endothelial cells common in head and neck. It's relatively rare in oral cavity [1] and uncommon in pharynx, particularly in the oropharynx [2]. About 60 %-70 % of all the hemangiomas are found in the head and neck region [1]. They can be divided in cutaneous, mucosal, intramuscular or intra-osseous [3]. Hoarseness, dysphagia and dyspnea are the most common symptoms. Moreover, this kind of lesion is related to high risk of bleeding and consequent serious complications [2].

On macroscopic examination, haemangiomas are characterized by soft or cystic tissue and blue-red mucosa. Ulceration, necrosis or chronic infection can be detected in large lesions [2]. Non-specific clinical and radiological features of the disease make diagnosis a challenge [4].

In literature, several treatments are reported including oral corticosteroids, intralesional injection of sclerosing agents, interferon a-2b, radiation, electrocoagulation, cryosurgery, laser therapy, embolization, and surgical excision of the hemangioma [5].

We present the clinical case of a 72 years-old patient affected by a huge neoformation in the tongue base suspected to be a schwannoma and treated with a new transoral technology. This work has been reported in line with the SCARE criteria [6].

## 2 Case report

**Fig. 1:**
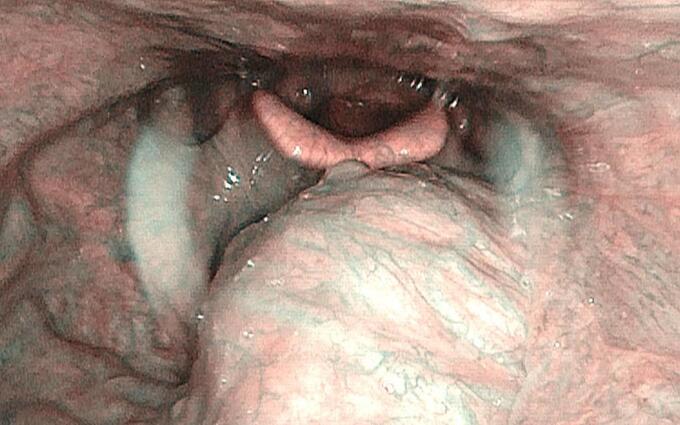
Submucosal bulging neoformation in the base of the tongue extending from the left beyond the midline, with rich vascular network (narrow band imaging image).

A 72 years-old man came to our ENT Clinic to perform a pre-operative evaluation of laryngeal morphology and motility for total thyroidectomy surgery. Patient was affected by high blood pressure pharmacologically treated and had no history of smoking or alcohol abuse. So, during the examination, flexible laryngoscopy showed a submucosal bulging and non-bleeding neoformation, about 2–3 cm in diameter, at the base of the tongue extending from the left beyond the midline. Its overlying mucosa had a rich vascular network (Fig. 1). Glosso-epiglottic valleculae were normo-explorable. Larynx was normal for both for motility and morphology. At the oropharyngoscopy this lesion was not visible, the motility of the tongue was preserved such as the oropharyngeal reflexes. No palpable neck lymphadenopathy was detected. The patient didn't report related symptoms such as dysphagia or dyspnea, the sense of taste was also preserved.

Neck computed tomography (CT) with contrast medium was performed: a suspected malignant lesion on the left hemisection of the tongue base, with non-homogeneous contrast enhancement was detected (maximum size of 4 cm × 4 cm × 3 cm approximately). No abnormalities or lymphadenopathies were detected on CT scan.

**Fig. 2:**
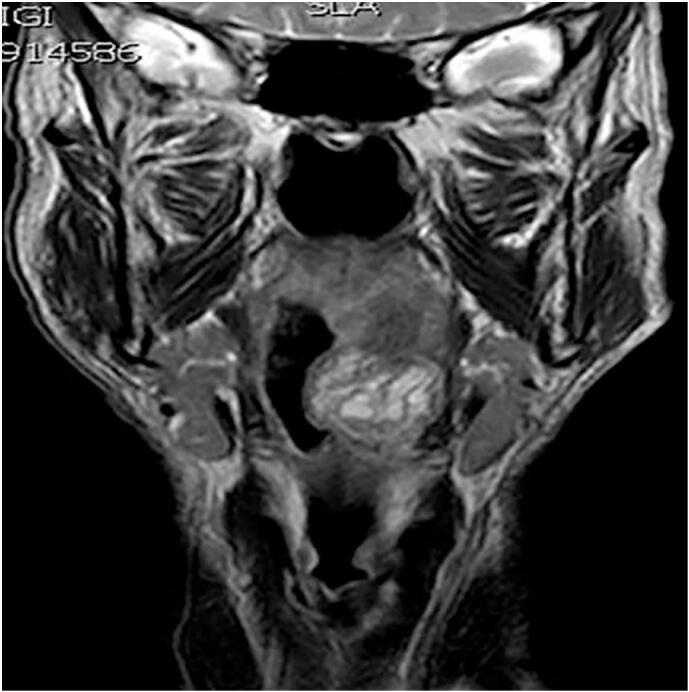
High intensity with hypointense areas at T2 MRI: coronal scan.

In order to better study this lesion before surgery, also a neck magnetic resonance imaging (MRI) with paramagnetic contrast medium was performed. The exam showed an oval neoformation in the left half of the tongue base, between the genioglossus and hyoglossus muscles, with regular margins, that moved back the epiglottis and caudally the left glosso-epiglottic vallecula and caused an asymmetrical narrowing of oropharyngeal lumen. Moreover, on MRI, the lesion showed homogeneous intensity - predominantly medium-low intensity with hypointense and hyperintense areas - at T1 and - predominantly high intensity with hypointense areas - at T2 and STIR (Short-TI Inversion Recovery), restricted diffusion on DWI (diffusion-weighted imaging), high ADC (Apparent diffusion coefficient), and progressive enhancement post contrast medium (maximum size 3.6 cm × 3.1 cm × 2.8 cm) (Fig. 2).

Based on these features, a schwannoma on the left half of the tongue base was hypothesized by radiologist.

Thus, the patient underwent surgery under general anesthesia: due to the hypervascularization of the lesion, left lingual artery ligation and tracheotomy were performed. In particular, the tracheotomy was performed to secure the airways. Then, a senior head and neck surgeon performed a complete excision of this neoformation: after placement of the Jennings Mouth Gag, the surgeon performed transverse incision of bilateral genioglossus and palatoglossus muscles for greater freedom of movement and therefore better exposure of the base of the tongue. At the same time, the maximum protrusion of the tongue was obtained by a traction point on the body of the tongue with a silk stitch. Once the lesion on the tongue base was visualized, a partial glossectomy was performed transorally using Thunderbeat®. No bleeding or other peri-operative complications occurred. A nasogastric tube was placed and removed on 5th day after surgery. The tracheotomy cannula was removed on the 6th day.

**Fig. 3:**
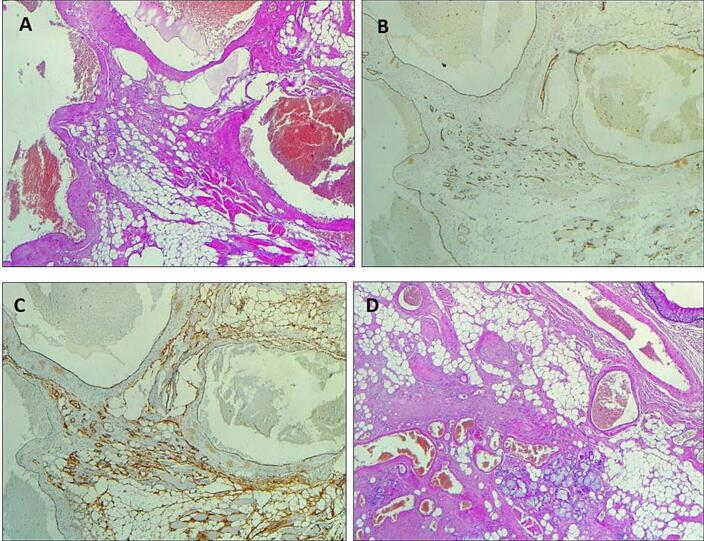
Immunohistochemical analysis: lesion comprised of dilated congested thin walled vessels, with thick feeding vessels at periphery (a), CD31 (PECAM1): cytoplasmic and membranous positivity; highly sensitive and very specific for vascular differentiation (b), CD34: cytoplasmic and membranous positivity; less specific (c), lobules of capillary sized vascular channels, lined by single layer of flattened endothelial cells (d).

Histopathologic analysis revealed that the lesion was a cavernous hemangioma of the base of tongue (measuring 4.3 × 3.5 × 2.5 cm) covered by pluristratified squamous epithelium with regular architecture and multiple medium and large vessels, most often ectasic and congested, sometimes cystically dilated, in the chorion. Immunohistochemical positivity for CD31 and CD34 confirms the vascular nature of the lesion. Resection margins were free (Fig. 3).

**Fig. 4:**
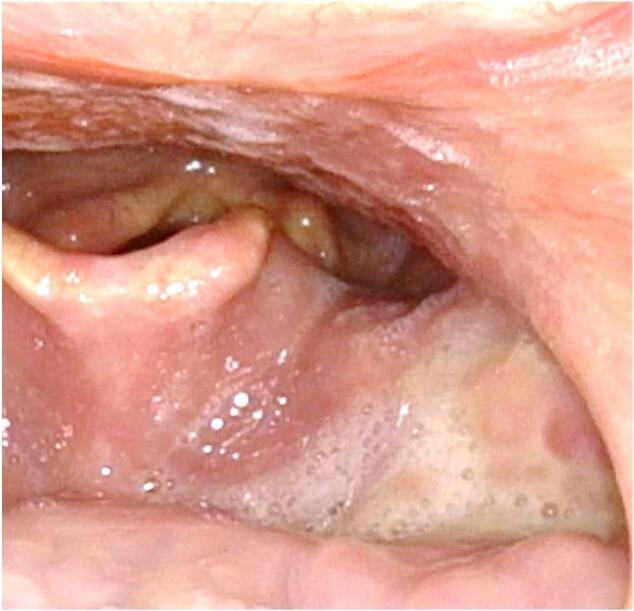
One month post-op.

In the post-operative, patient underwent follow-up and today, one year after surgery, he doesn't show sign of disease relapse (Fig. 4).

## 3 Discussion

According to Mulliken and Glowacki, vascular malformations of the tongue are divided into hemangiomas and vascular malformations. Hemangiomas are usually not present at birth; they undergo rapid volumetric increase and subsequent involution and arise in 60 % of cases in the head-neck district. Vascular malformations, on the other hand, are present at birth, grow in proportion to body size, and may degenerate or become hypertrophic [7].

Hemangiomas are the most common benign tumors of the head and neck in children and primarily affect the face, oral mucosa, lips and tongue [1,8].

According to the literature, the base of tongue is an extremely rare site of involvement.

Involvement of the tongue should be cautioned because of its susceptibility to masticatory trauma and subsequent bleeding and ulceration, difficulty swallowing and, in case of upper airway obstruction, dyspnea [9].

Hemangiomas are known to have a higher prevalence in women (3:1 to 7:1 predilection) and occur more frequently in infants and childhood, although some cases develop in adults [10].

They usually develop 2–4 weeks after birth with a rapid growth until the age of 6–8 months. Then they grow slowly and regress spontaneously in 70 % of cases by the age of 5–8 years [11].

There is no real evidence regarding the pathophysiology of hemangioma. It is thought that monocytes, considered the potential progenitors of endothelial cells in hemangioma, may be the key players in the onset of this neoplasm associated with genetic factors. Specifically, an imbalance in angiogenesis related to substances such as vascular endothelial growth factor (VEGF) and basic fibroblast growth factor (BFGF) is proposed to result in uncontrolled proliferation of vascular elements [1].

Histologically, hemangioma can be divided into capillary and cavernous forms [1], based on the size of vascular spaces [3]. Capillary hemangioma is characterized by a connective tissue stroma that supports small capillaries aligned by a single layer of endothelial cells, whereas cavernous hemangioma is formed by a thin layer of connective tissue septa lining sinusoids or large, thin-walled vessels [1]. Clinically, hemangiomas present as a soft, smooth or lobulated, sessile or pedunculated swelling and can range in size from a few millimeters to several centimeters. Upon pressure, the tumor lightens and generally varies in color from pink to purplish-red. Spontaneously or as a result of minor trauma, bleeding may occur [3].

Fourteen cases of cavernous hemangioma of the tongue have been described in the literature. Among them, only one interesting the base of tongue, like our case [12]. 71 % of the patients were female and 57 % were under 20 years of age. Only 14 % were over 60 years old. The most affected area of the tongue was the lateral surface, followed by the anterior and ventral surfaces. 92 % had a single lesion [13]. Except for six cases (42 %) that were asymptomatic, the most frequently reported symptoms were difficulty swallowing, pain, bleeding and, in two cases of giant hemangioma, dyspnea [14,15]. 78 % underwent surgery with complete removal of the lesion.

According to the literature, our case presents unusual features in several aspects: age, sex of the patient, and location of the tumor. In fact, male sex, age over sixty years and, especially, involvement of the base of the tongue are extremely rare clinical presenting features of a cavernous hemangioma.

Comparing our case with the only other example of hemangioma of the base of tongue reported in the literature [12], Beghdad et al. described a case of an adult female patient, while our patient is a male. Moreover, the two neoformations, measuring approximatively 4 cm in diameter, located in the same anatomical region, present a different clinic: in our case asymptomatically, in the other with chronic and persistent dysphonia associated with progressive dysphagia. Both were surgically removed, although with different techniques: we preferred a trans-oral approach with the Thunderbeat® after ligation of the lingual artery ipsilateral to the lesion to prevent intra-operative bleeding, Beghdad et al. used external approach with cervical incision. In both cases, pre-operative examinations did not allow to reach the correct diagnosis and both patients required a prophylactic tracheostomy. Patients, at follow-up, were free of disease. Gupta et al. reported a case of an adult woman affected by a cavernous hemangioma for about 20 years that involved the right side of the mobile tongue. In this case, the surgeon preferred a conservative approach, that was the sclerotherapy [16]. Due to the huge size of the lesion, the injection of sodium tetra decyl sulfate was repeated four times. On the contrary, in our case, the lesion affected the base of tongue and was treated by a one stage surgery, without post-operative complications.

In the diagnostic workup, radiological study of the lesion by CT and/or MRI is essential. Hemangioma appears on CT as a tumor of tissue density, with a weak enhancement pattern. On MRI it appears homogeneously hyperintense in T2-weighted images and is usually isointense to muscle in T1-weighted images, with significant enhancement after contrast administration [17]. This radiographic finding may lead to an incorrect assessment of the mass. In fact, the same imaging features are present in schwannomas [18], which is why, in our case, the radiologist had assumed it as a possible diagnosis. Schwannomas, or neurilemmas, are benign neoplasms arising from Schwann cells surrounding peripheral, cranial, or autonomic nerve sheaths. They are solitary lesions without genetic or gender predisposition, involving the head-neck region in 25–48 % of cases in patients aged 20–50 years. When the tongue is involved, schwannomas usually present as a painless mass. The presence of S-100 protein in the immunohistochemical study is a classic marker for diagnostic confirmation, that was not expressed in our lesion [18].

Several factors, including the age of the patient, the size and extent of the lesions, and their clinical features determine the management and treatment of hemangiomas. Several treatments are described in literature for the management of hemangiomas: oral corticosteroids, intralesional injection of sclerosing agents, interferon a-2b, radiation, electrocoagulation, cryosurgery, laser therapy, embolization, and surgical excision [5]. Recurrence has been reported; however, one year after surgery, our patient doesn't show sign of relapse [3]; indeed, after the transoral removal, the resection margins resulted free of lesion. Surgery allows to achieve a definitive excision of the mass by a single step. On the contrary, as reported also by Gupta et al. [16], conservative therapies, such as oral steroids or local injection of drugs, result longer and require more treatment cycles, until the complete – or almost – remission of the lesion. Obviously, this may correlate with higher risk of recurrence. So, considering the strengths and the flaws of these procedures, we performed a transoral surgery through the Thunderbeat® device in order to ensure higher probability of radicality with minimal invasiveness.

Indeed, transoral ultrasonic surgery (TOUSS) with the Thunderbeat® belongs to the minimally invasive transoral surgical techniques (MIS) along with electrocautery, transoral laser microsurgery (TOLM) and transoral robotic surgery (TORS) [19]. The Thunderbeat® is the first surgical instrument that combines high-frequency bipolar energy and ultrasonic technology while achieving two results simultaneously: in fact, it cuts quickly and precisely and dissects tissue and seals vessels up to 7 mm in diameter simultaneously [20]. Initially used for laparoscopic, urologic and gynecologic surgery, it was introduced, by Fernández-Fernández et al., in transoral surgery, achieving the same results as the daVinci surgical system (Intuitive Surgical, Sunnyvale, CA) at low cost [21]. So, based on the advantages of this surgical device and on the clinical and radiological characteristics of the lesion, it was decided to use the Thunderbeat® to ensure greater intra- and post-operative safety in terms of bleeding and radicality of the excision.

With the radiological examination alone, ligation of the lingual artery could have been an excessive maneuver, but considering the histological outcome, it turned out to be an adequate prophylactic procedure. This underlines the importance of a complete study of the pathology in the pre-operative period with a multidisciplinary team to achieve the best possible therapeutic approach.

## 4 Conclusion

This case report would like to emphasize two important messages: first, in the presence of a detected, richly vascularized tongue base lesion, without clear imaging features, it must be taken into account that it may be a cavernous hemangioma of the tongue base, although it's rare. Moreover, the use of the Thunderbeat® makes possible to treat lesions transorally with minimal risk of bleeding and no cosmetic damage, achieving the same advantages as TORS (not present in most ENT units) with lower costs and shorter learning curve.

## Consent

Written informed consent was obtained from the patient for publication and any accompanying images. A copy of the written consent is available for review by the Editor-in-Chief of this journal on request.

## Ethical approval

We acquired the consent for publication from patient but we don’t require ethical approval since it is a anonymous case report.

## Funding

This research did not receive any specific grant from funding agencies in the public, commercial, or not-for-profit sectors.

## Author contribution

Carmelo Saraniti: concept of design, supervision, validation of final version

Barbara Verro, Davide Burrascano, Iara Comincini: data collection and analysis, writing the paper

Anna Martorana: validation of the final version

## Guarantor

Barbara Verro.

## Conflict of interest statement

The authors declare no conflict of interest.
